# A87 LEVERAGING DIGITAL HEALTH: SYMPTOM TRACKING IN PEDIATRIC IBD USING A SMARTPHONE APP FOR ENHANCED PATIENT-REPORTED OUTCOMES

**DOI:** 10.1093/jcag/gwae059.087

**Published:** 2025-02-10

**Authors:** É L’Heureux-Hubert, T Mah, M Fleur-Aimé, P Louis, C Vaccarino, H Khouna, R Martin, R Kubinski, L Chapuy, L Cuccia, C Deslandres, P Jantchou

**Affiliations:** Gastro-entérologie, Centre hospitalier universitaire Sainte-Justine, Montréal, QC, Canada; Gastro-entérologie, Centre hospitalier universitaire Sainte-Justine, Montréal, QC, Canada; Gastro-entérologie, Centre hospitalier universitaire Sainte-Justine, Montréal, QC, Canada; Gastro-entérologie, Centre hospitalier universitaire Sainte-Justine, Montréal, QC, Canada; Gastro-entérologie, Centre hospitalier universitaire Sainte-Justine, Montréal, QC, Canada; Gastro-entérologie, Centre hospitalier universitaire Sainte-Justine, Montréal, QC, Canada; injoy technology pteltd, Montréal, QC, Canada; injoy technology pteltd, Montréal, QC, Canada; Gastro-entérologie, Centre hospitalier universitaire Sainte-Justine, Montréal, QC, Canada; injoy technology pteltd, Montréal, QC, Canada; Gastro-entérologie, Centre hospitalier universitaire Sainte-Justine, Montréal, QC, Canada; Gastro-entérologie, Centre hospitalier universitaire Sainte-Justine, Montréal, QC, Canada

## Abstract

**Background:**

Patient-reported outcome measures (PROMs) are essential for managing chronic diseases, as they provide valuable insights into patients’ perspectives on their condition’s impact and help tailor treatments accordingly. However, collecting this information from pediatric patients with inflammatory bowel disease (IBD) presents several challenges, such as the reluctance of patients to discuss sensitive topics such as bowel movements, defecation, and rectal bleeding. Additionally, the subjective nature of physicians’ evaluations compared to the experiences of patients affect the symptom assessment. An IBD app could help teenagers to routinely assess the clinical symptoms.

**Aims:**

The objectives of the study were twofold: first, to describe the trajectory of symptoms as self-reported by patients through a digital application, and second, to compare these symptoms with those evaluated by physicians during medical visits.

**Methods:**

We conducted a prospective study involving teenagers with IBD aged 15-18 years old. All participants had a smartphone and an understanding of English. They received an app (Injoy Gut Health tracking by Phyla Technologies Inc.) that allowed for the collection of symptoms at home, which they were asked to use daily. At inclusion, participants completed the IBD Control questionnaire. Additionally, we collected information on disease status such as the physician global assessment (PGA).

**Results:**

We included 25 children (60% female) with a median age at diagnosis of 14.5 years (interquartile range (IQR): 13.2-14.5) for IBD (64% Crohn’s disease) for a median of 3.6 years (IQR: 2.7-5.4). Most participants (84%) were in complete remission and 16% had mild active disease, according to the PGA. The teenagers completed the app survey for a median of 122 (IQR: 44-200) days.

Participants reported gastrointestinal symptoms during daily data collection: 48% experienced rectal pain, 64% experienced nausea, 96% experienced gas, 76% experienced bloating and 80% experienced abdominal pain. Finally, 28% noted blood in their stools representing a median of 7.8% (IQR 8.88-11.9) of their stools. The PROMs filled at baseline depicted a spectrum of disease activity. The median IBD-control-8 subscore was 12 (IQR: 10-15).

The PROMs and the data self-collected daily at home were not in agreement with the PGA. Indeed, symptom data showed that participants experienced a median of 7 (IQR: 4-8) symptoms while 92% (23) of patients reported more than two symptoms.

**Conclusions:**

These results highlight the importance of incorporating PROMs for more precise and personalized management of IBD in children. The use of an app for at-home daily data collection proved to be a valuable tool in capturing real-time symptomatology.

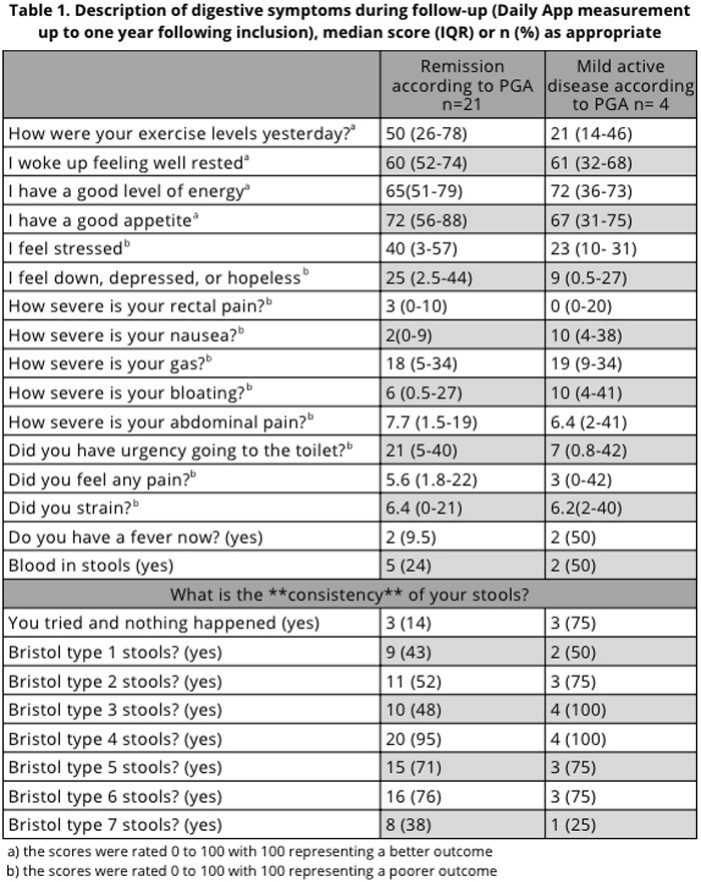

**Funding Agencies:**

